# Influence of Environmental Tobacco Smoke and Air Pollution on Fetal Growth: A Prospective Study

**DOI:** 10.3390/ijerph17155319

**Published:** 2020-07-23

**Authors:** Meiman Maggie Chen, Chun-Hui Chiu, Chi-Pin Yuan, Yen-Chi Liao, Su-Er Guo

**Affiliations:** 1 Department of Nursing and Graduate Institute of Nursing, College of Nursing, Chang Gung University of Science and Technology (CGUST), Chiayi County, Puzi 613016, Taiwan; mmchen@mail.cgust.edu.tw; 2 Chronic Diseases and Health Promotion Research Center, Chang Gung University of Science and Technology (CGUST), Chiayi County, Puzi 613016, Taiwan; 3 Research Center for Chinese Herbal Medicine and Research Center for Food and Cosmetic Safety, Graduate Institute of Health Industry and Technology, College of Human Ecology, Chang Gung University of Science and Technology, Taoyuan 333324, Taiwan; chchiu@mail.cgust.edu.tw; 4 Department of Nursing, Ditmanson Medical Foundation Chiayi Christian Hospital, Chiayi County, Chiayi 600566, Taiwan; 01454@cych.org.tw (C.-P.Y.); 12262@cych.org.tw (Y.-C.L.); 5 Division of Pulmonary and Critical Care Medicine, Chang Gung Memorial Hospital, Chiayi County, Puzi 613016, Taiwan; 6 Department of Safety Health and Environmental Engineering, Ming Chi University of Technology, New Taipei 243303, Taiwan

**Keywords:** environmental tobacco smoke, air pollution, fetal growth, birth length, birth weight

## Abstract

Exposure to air pollution during pregnancy leads to adverse pregnancy outcomes. Few studies have evaluated the influences of air quality, including environmental tobacco smoke (ETS) and particulate matter (PM), on fetal development, which this study examined. This longitudinal correlation study used multiple linear regression data analysis of PM_2.5_/PM_10_, self-reported ETS exposure, urinary cotinine level, maternal characteristics, and birth parameters (gestational week, body weight, body length, head, and chest circumferences) with the effect of air quality on fetal growth. The study included 74 pregnant women (mean age 31.9 ± 4.2 years, body mass index 23.6 ± 3.8 kg/m^2^, average gestational duration 38.5 ± 0.8 weeks). ETS exposure decreased birth length by ≥1 cm, and potentially is an independent risk factor for fetal growth restriction, and pregnant women should avoid indoor and outdoor ETS. However, neither PM_2.5_/PM_10_ nor ETS was associated with low birth weight or small for gestational age. This study adds to the evidence base that ETS exposure of nonsmoking pregnant women affects the fetal birth length. Family members should refrain from smoking near expectant mothers, although smoking in the vicinity of their residential surroundings potentially exposes mothers and their fetuses to ETS. Public pollution and childbirth education classes should include details of indoor ETS.

## 1. Introduction

Exposure to air pollution affects pediatric health in many ways [1,2]. The most important stage of children’s health begins during fetal development. Thus, adverse pregnancy outcomes are important health indicators of neonatal and infant health. Exposure to toxicants during pregnancy has been linked to adverse pregnancy outcomes, which include shorter gestational duration [3,4,5,6] and fetal growth restriction [3,7,8,9]. Plausible mechanisms mediating the influence of toxicant exposure on a developing fetus may include impaired oxygen and nutrient transport to the fetus due to oxidative stress, inflammatory reactions, and hemodynamic changes because of prenatal exposure to air pollutants [10,11]. Fetal growth restriction is a significant pediatric health indicator because it is associated with neonatal morbidity and mortality [1,12,13]. Studies have found that fetal growth restriction may be attributed to outdoor air pollutants, including particulate matter (PM) with aerodynamic diameter of 10 or 2.5 µm (PM_10_ or PM_2.5_, respectively), sulfur dioxide (SO_2_), nitrogen dioxide (NO_2_), carbon monoxide (CO), ozone (O_3_) [4], and secondhand smoke [3].

Maisonet and colleagues [3], in their review of four investigations from the United States, China, the Czech Republic, and Korea on the impact of the average concentration of PM_10_, SO_2_, NO_2_, CO, and O_3_, found small (odds ratio (OR) = 1.04–1.22) to moderate (OR = 2.6) effects of the studied toxicants on low birth weight and fetal growth restriction. The OR (1.04–2.6) of term low birth weight, which was defined as birth weight below 2500 gm, and small for gestational age (SGA), which is defined as the neonate’s weight at birth, was below the 10th percentile with maternal exposure to PM_2.5_ of 35.6 µg/m^3^. In concordance with the report of Maisonet et al. [3], Jedrychowski and colleagues [14] conducted a study in Poland that enrolled 362 nonsmoking pregnant women who gave birth between 34 and 43 weeks of gestation and found that a median total personal PM_2.5_ exposure of 36.3 µg/m^3^ over a 48 hour period was associated with a 0.9 cm, 0.3 cm, and 128.3 g reduction in birth length, head circumference (HC), and birth weight, respectively; however, a significant interrelationship between environmental tobacco smoke (ETS) and PM_2.5_ could not be ascertained, although the analytical models with both ETS and PM_2.5_ showed a nonsignificant effect of ETS.

Ballester et al. [7] investigated a prospective birth cohort comprising 785 Spanish pregnant women and their newborns, and reported that NO_2_ exposure in the first trimester reduced birth length by 0.27 cm (confidence interval (CI): −0.51 to −0.03) and birth weight by 40.3 g (CI: −96.3 to 15.6); the OR of SGA by weight and length were 1.37 to 1.42, respectively. [7] Based on their analysis of a live-birth cohort of 3692 Chinese study participants, Yuan et al. [9] reported evidence that supported the previous findings that ambient air pollutants reduce birth weight and gestational length (preterm delivery (PTD)). They support that exposure to air pollutants in late pregnancy constitutes a crucial window of pollution exposure. However, based on an analysis of data from 1, 742, and 183 birth records, Kim et al. [6] found insignificant effects of PM_10_ >70 µg/m^3^ on low birth weight. Guo et al. [15] conducted a systematic review of 40 studies with case–control and cohort study designs, and reported results that supported previous findings: there was a small effect of air pollution on low birth weight (OR = 1.03–1.21). A literature review clearly shows that air pollution affects fetal growth, although its effect size is small.

Active maternal smoking is known to affect the health of children [16,17,18]. However, there are inconclusive findings with regard to the birth outcomes of non-smoking pregnant women with exposure to smoke [17,18,19,20]. Based on their systematic review and meta-analysis of 23 prospective studies, Leonardi et al. [20] reported that maternal exposure to environmental tobacco smoke (ETS) reduced mean birth weight by 33 g and conferred a 32% increased risk of low birth weight of less than 2500 g. Similarly, Salmasi et al. [19] found that ETS-exposed women (*N* = 48,439) had a 16% higher risk of their baby’s birth weight being less than 2500 g than women without ETS exposure (*N* = 90,918). In Taiwan, the proportion of female adults who smoked decreased from 5.3% to 2.8% from 2004 to 2006 [17]. However, in the same time period, the proportion of paternal smoking during a spouse’s entire pregnancy was almost 50%, which was similar to the proportion reported for the last 10 years [17]. Thus, approximately half of the pregnant women were exposed to passive smoking at home. The findings of Ko et al. support previous reports of the effect of maternal smoking on fetal growth but did not find evidence of paternal smoking on fetal growth [17]. It may be that fathers did not smoke in the presence of pregnant mothers. It is unknown whether pregnant women may be exposed to indoor ETS at places other than their home. A literature review indicated that air pollution, in spite of a small effect size, affects fetal growth. The findings on the effects of ETS exposure of nonsmoking women were inconclusive. Air pollutants that comprise ETS might increase the risk of fetal growth retardation. Therefore, we hypothesized that outdoor ambient air pollutants and ETS affect fetal growth. The aims of this study were to examine the effects of PM and ETS on fetal development among pregnant women.

## 2. Materials and Methods

### 2.1. Study Design

This prospective study involved data collection between March 2017 and September 2019 at two time points during each pregnancy. Ethics approval (certificate no: 106059) for study conduct was obtained from the Institutional Review Board of Ditmanson Medical Foundation Chia-Yi Christian Hospital, and our research work was undertaken in accordance with ethical principles of autonomy and confidentiality at all times. The anonymity of study participants was ensured with the use of a study identification number without any personal identifiers.

### 2.2. Participants

Purposeful sampling was used to recruit the study participants. The inclusion criteria were: (1) singleton pregnancy of 12–16 weeks; (2) age of 20–45 years; (3) ability to read Chinese and speak Mandarin; and (4) residence in Chaiyi (based on postal code) for at least 6 months. We excluded women who smoked after pregnancy confirmation or had pregnancy-related complications, such as placenta previa, gestational diabetes mellitus, pregnancy-induced hypertension, cardiac disease, and premature rupture of membranes. No previous studies have reported an association between maternal urinary cotinine concentrations and fetal growth parameters. Therefore, assuming that the body length of the newborn was 47.5 cm in the none-to-low cotinine group and 49 cm in the moderate-to-high cotinine group with a common standard deviation of 2 cm, a minimum sample size of 78 subjects was required to achieve an alpha level of 5% and a power level of 90%.

### 2.3. Measurements and Instruments

The two time points of data collection were: (1) at 12–16 weeks of gestation and (2) at the birth of the baby. Data collection of ETS exposure was obtained from self-reported questionnaires and laboratory confirmation of cotinine in urinary sample at each time point.

#### 2.3.1. Demographic Characteristics

The questionnaire comprised four sections. In the first section, personal details, including age, weight, height, last menstruation date (LMP), estimated date of delivery (EDD), parity, education level, employment, insurance type, and religion, were collected. The second section contained questions on women’s pre-pregnancy ETS status, ETS exposure at home during pregnancy, and ETS exposure in their place of employment and in public places within the previous 30 and 7 days, respectively. A sample question is as follows: In the past 30 days, how often were you exposed to ETS: “never”, “occasionally (1–2 days/week)”, “sometimes (3–4 days/week)”, “always (5–6 days/week)”, or “everyday”. The third section collected ultrasonographic measurements, such as crown–rump length (CRL), biparietal diameter (BPD), head circumference (HC), femur length (FL), abdominal circumference, and birth weight and length.

#### 2.3.2. Fetal Growth Measurements

The CRL was used as a baseline fetal growth indicator in the first trimester, as it is the most accurate method to confirm gestational age, especially if the report of the woman’s LMP is unreliable. Prior to 14 weeks of gestation, the CRL is accurate to within 5 to 7 days. Beyond 14 weeks of gestation, the CRL was not used; instead, the BPD, HC, abdominal circumference (AC), and FL were measured at 20–21 weeks of gestation to estimate fetal growth. The BPD most accurately corresponds to gestational age, with a variation of 7–10 days after the second trimester, whereas the HC may be influenced by the shape of the head. The FL correlates with BPD and gestational age, and AC is the most responsive indicator of fetal growth. Birth weight and length were recorded at birth. Fetal growth was considered SGA if the baby’s weight was below the 10th percentile of reference measurements after 37 weeks of gestation. Alternatively, the term low birth weight was used to assess fetal growth restriction if the baby’s birth weight was less than 2500 g.

#### 2.3.3. Environmental Tobacco Exposure

Nicotine is a biomarker of cigarette smoking. Cotinine is a metabolic byproduct of nicotine and 70–80% of absorbed nicotine is estimated to be converted into cotinine. Serum, urinary, and salivary levels of cotinine have been measured to assess ETS exposure. Because the average half-life of cotinine in the serum, urine, and saliva is nearly similar [21], we decided to use noninvasive methods to measure cotinine in the urinary sample in addition to the self-reported ETS exposure questionnaire to correlate passive smoke exposure [22,23]. The content of cotinine in human urine by using ultra performance liquid chromatography-tandem mass spectrometry was ascertained in accordance with the method described by Xu et al. [24]. The self-reported ETS exposure questionnaire we used was to ascertain the participant’s exposure at home and in their place of employment and in public places in the previous 30 and 7 days, respectively.

#### 2.3.4. Air Quality Assessment

Data on the concentration of PM_2.5_/PM_10_ was collected from the Taiwan Air Quality Monitoring Network (TAQMN) and calculated from the hourly PM_2.5_/PM_10_ monitoring records of the Yun-Chia-Nan air-quality region. The TAQMN, established by the Taiwan Environmental Protection Administration (EPA), R.O.C. in 1993, had 76 stationary monitoring stations distributed throughout the island in 2015 [25]. According to geographical characteristics and air-quality conditions, the TAQMN divided Taiwan into seven air-quality regions: Northern, Chu-Miao, Central, Yun-Chia-Nan, Kao-Ping, Yilan, and Hua-Tung. PM_2.5_/PM_10_ concentrations in Taiwan have been measured continuously since 2006 [25]. Exposure of PM_2.5_/PM_10_ during pregnancy were defined as averages of monthly level of PM_2.5_ from one month before the last menstruation date (LMP) to delivery date.

#### 2.3.5. Statistical Analysis

Data analysis included four items of subjective exposure frequency to smoke during pregnancy. We obtained the factor score of each maternal parameter using the exploratory factor analysis, which included only one factor. The relationship between continuous variables of exposure (i.e., urinary cotinine level) and neonatal parameters was assessed using the Pearson correlation or Spearman rank correlation (for urinary cotinine, due to the non-normal distribution). The relationship between categorical variables of exposure (i.e., none, low, and high levels of urinary cotinine) and neonatal parameters were tested using the independent sample t-test or one-way analysis of variance. The birth length among different urinary cotinine levels was further compared in each stratum of the other exposure variables (i.e., subgroup of PM_2.5_ by the median value) by using one-way analysis of variance. Finally, the association of urinary cotinine level with birth length was investigated by linear regression analysis, with and without possible confounders. Several possible maternal confounders, including age and body mass index (BMI), subjective frequency of smoke exposure during pregnancy, nutritional supplements, and alcohol intake were adjusted in the multivariate linear regression model. All tests were two-tailed and a *p*-value <0.05 was considered indicative of statistical significance. Data analyses were conducted using SPSS 25 (IBM SPSS Inc, Chicago, IL, USA).

## 3. Results

### 3.1. Characteristics of the Maternal Participants and Neonates

This study included 74 pregnant women. Of the 74 participants, 61 (82%) women had a vaginal birth whereas 13 (18%) women had a planned cesarean section. The mean age was 31.9 years (standard deviation (SD = 4.2 years) with an average education level of 15.4 years (SD = 1.7 years). The mean body weight was 60.9 kg and the BMI was 23.6 kg/m^2^, respectively. Most (89.6%) of the study participants consumed nutritional supplements whereas a few (3%) reported alcohol intake during pregnancy. Approximately half (48.6%) of the family members of the study participants smoked before their pregnancy, whereas only a few (36.5%) of the study participants perceived an exposure to passive smoking from their partner and family members during their pregnancy. The scores for the items on subjective exposure frequency to smoke ranged from 2 to 3. The average levels of urinary cotinine was 4.7 ng/mL at birth, and 7 (9.5%) of the participants had no residual urinary cotinine. The average PM_2.5_ and PM_10_ levels in the previous year were 28.0 and 48.4 μg/m^3^, respectively (Table 1).

All infants had a normal Apgar score at 1 and 5 min (9 and 10, respectively) after birth. In addition, all infants had a normal appearance. With regard to the neonatal parameters, the average gestational age was 38.5 weeks, with a low variation (SD = 0.8 weeks). The mean body weight was 3033 g (SD = 317 g). The HC, birth length, and chest circumference were 33.4, 48.5, and 32.1 cm, respectively (Table 1).

### 3.2. Relationships Between Exposure Variables of Interest and Neonatal Parameters

The correlation analyses showed that there were no significant relationships between all of the continuous variables of exposure (both PM_2.5_/PM_10_ and ETS) and neonatal parameters (*r* = 0.02–0.16, *p* > 0.05). In further analyses, we categorized the continuous variables of exposure. The factor score of subjective frequency of smoke exposure during pregnancy was used to equally divide the study population into three subgroups. Based on the urinary cotinine level, the study population was divided into the “none”, “low”, and “high” subgroups (with equal number of participants in the latter two subgroups). Similarly, based on the PM_2.5_ and PM_10_ levels, the study population was equally divided into two subgroups. The results suggested that the birth length significantly differed among subgroups with different urinary cotinine levels (*p* = 0.049). In the multivariate comparison, the birth length was significantly shorter in the high cotinine subgroup than in the low cotinine subgroup (*p* < 0.05). Moreover, the birth length was significantly shorter in the high urinary cotinine subgroup than in the none and low urinary cotinine subgroups combined (*p* < 0.05; Table 2).

We further compared the birth length among different urinary cotinine levels in each stratum of other exposure variables (i.e., subgroup of PM_2.5_ by the median value). We observed that the average birth length was more than 48 cm in the none and low urinary cotinine subgroups regardless of the levels of other exposure variables. Moreover, the average birth length was less than 47 cm in the high urinary cotinine subgroup regardless of the subgrouping by the other exposure variables (Table 3). These results implied the absence of an additive effect between the urinary cotinine level and other exposure variables.

Furthermore, linear regression analysis revealed that high urinary cotinine level was associated with a significantly shorter birth length (regression coefficient = −1.1 cm, 95% confidence interval = −2.3 to −0.001 cm, *p* = 0.049). This effect persisted after adjusting for several confounders (regression coefficient = −1.4 cm, 95% confidence interval = −2.4 to −0.3 cm, *p* = 0.012; Table 4).

## 4. Discussion

To the best of our knowledge, this study constitutes the first investigation of urinary cotinine to assess the influence of ETS on fetal growth. The potential mechanisms underlying the effect on birth growth is that trans-placental exposure to ambient air pollutants and ETS could trigger an oxidative stress process that may lead to DNA damage and fetal growth restriction [10,26]. There is inconsistent evidence of the effect of ETS on fetal growth. We investigated the association between birth outcomes (e.g., birth weight and birth length), SGA, and early gestational exposure to ETS and ambient PM_2.5_/PM_10_. The analysis of urinary cotinine levels, after controlling for potential confounders (i.e., maternal age, BMI, subjective frequency of smoke exposure during pregnancy, nutritional supplements, and alcohol intake; *p* = 0.012), showed that high levels of cotinine exposure during pregnancy significantly reduced birth length, but had an insignificant effect on the birth weight of term infants (Table 4). Our results are partly consistent with the reports in the literature [14,19,20] but differ from that of a Taiwanese study by Ko et al. [17], who reported that paternal smoking at home did not increase the OR for the decline in birth measurements. The possible reasons for the differences in the results between the present study and the one by Ko et al. [17] include the definition, scope, and measurement of ETS. In the present study, ETS was defined as passive smoking regardless of exposure at home or any other places. Moreover, we used urinary cotinine levels as an objective indicator of ETS. In addition, compared to non-exposure during pregnancy, high-level cotinine exposure during pregnancy reduced the birth length by 1.4 cm, on average. This reduction is slightly higher than that reported (reduction of 0.90 cm vs. 1.06 cm) in the study by Jedrychowski et al. [14] and Krstev et al. [27], but is supported by two studies in Spain. Ballester et al. [7] conducted a prospective cohort study that included 785 mother–newborn pairs to ascertain the effect of prenatal NO_2_ exposure on fetal growth; they found that exposure to NO_2_ levels > 40 μg/m^3^ during the first trimester was associated with a change in birth length of −0.27 cm (95% confidence interval (CI) −0.51 to −0.03) and with a change in birth weight of −40.3 g (−96.3 to 15.6). Moreover, a 10 μg/m^3^ increase in NO_2_ exposure levels during the second trimester was associated with neonates being SGA by birth weight (OR 1.37 (1.01–1.85)), whereas the OR for being SGA by birth length was 1.42 (0.89–2.25). Unlike Ballester et al. [7] who evaluated a single ambient air pollutant, Estarlich et al. [28] investigated the association of ambient NO_2_ and benzene with fetal growth and found that an increase of 10 µg/m^3^ in NO_2_ exposure during pregnancy was associated with a birth-length change of −0.9 mm (95% CI −1.8 to −0.1 mm). The findings from this study may contribute a new perspective for future studies on ambient pollutants that affect the fetal body length. Our results are supported by an appropriate cross-validation of the research process and the outcomes. Firstly, all infants were normal in appearance on the physical examination conducted immediately after birth, wherein we ruled out Down’s syndrome, which may be associated with a shorter birth length. Secondly, no participants were identified as being at high risk of having trisomy 21. Thirdly, we excluded women with pregnancy-induced hypertension, which can compromise placental blood supply and consequently affect fetal growth. The absence of this effect in all infants was indicated by the normal Apgar scores (9 and 10 at 1 and 5 min, respectively, after birth). Nevertheless, unlike in Jedrychowski et al.’s [14] and Krstev et al.’s [27] study, this study did not find any evidence of a reduction in the HC.

The findings of this study showed that there was no significant relationship between air quality (both PM_2.5_/PM_10_ and self-reported exposure to smoke) and birth weight. One possible reason for this finding might be maternal nutrition, which we could not evaluate because of inadequate sample size and the many participants with missing data. Further studies need to investigate the correlation of birth weight with the exposure to air pollutants, maternal nutrition, and nutritional supplements as potential confounders. The small sample size of this study may be another reason for the finding with regard to birth weight. A study [14] that enrolled 362 pregnant women found that air pollution was associated with fetal growth restriction. Thus, given the limited literature on this relationship, further studies with larger sample sizes are warranted. Moreover, in this study, there was no birth weight below the 10th percentile or less than 2500 g (low birth weight at term). Furthermore, 90% of our study participants were on at least one nutritional supplement (e.g., comprehensive vitamins, probiotics, calcium tablets, lecithin, etc.) and attained the recommended pregnancy weight gain (range: 11.5–16 kg for women with BMI in the normal range: 19.8–26; weight gain in study participants, mean ± SD: 11.66 ± 4.37 kg) based on the nutritional guidelines for pregnancy [29].

Prenatal exposure to PM_2.5_/PM_10_ was unassociated with fetal growth in this study. There are probable reasons for the absence or small effect of our results. Firstly, the study participants may not have been exposed to markedly high levels of PM_2.5_/PM_10_ concentrations during their pregnancies (28.0 ± 2.3 vs. 48.4 ± 5.2, respectively; Table 1). In addition, 60 women (90%) consumed nutritional supplements during their pregnancy. Nutritional supplements potentially have an antioxidative effect, which may offset the toxic effects of air pollutants on a growing fetus. Studies in Poland [30,31] found that vitamin A supplementation and fish oil consumption decreased the likelihood of fetal growth restriction due to exposure to air pollutants during pregnancy.

With regard to whether outdoor ambient air pollutants and ETS affect fetal growth, the results of this study indicated that there was no additive effect between the urinary cotinine level and exposure to PM_2.5_/PM_10_. It is possible that there was no marked exposure to abnormal PM concentrations in the long term among our study population, although they may have been exposed to short-term abnormal PM_2.5_/PM_10_ if we considered the PM_2.5_/PM_10_ concentrations level per day and not per month. Nonetheless, there were only 47 days (12.88% of the year) with PM_2.5_ above 35 µg/m^3^. Some studies [3,14] have revealed a significant association between birth outcomes and abnormal PM_2.5_ exposure (PM_2.5_: 35.6–36.3 µg/m^3^). However, the abovementioned previous studies did not evaluate ETS or undertake objective measurements. Thus, the possible influence of toxicant exposure during pregnancy is a vital issue that is a concern for healthcare professionals, and further research in well-designed case–control studies should be undertaken in areas with abnormal and normal PM concentrations. In addition, smoking in front of expectant mothers may expose them to ETS. The ETS rate of pregnant women during their pregnancy in Taiwan increased from 75.84% in 2012 to 85.65% in 2016, based on a government regular survey [32,33]. Public pollution and childbirth education should include strategies to counter indoor ETS.

This study has limitations that should be acknowledged. Firstly, the data collection from a single center with convenience sampling could limit the generalizability of the findings. However, our population characteristics are representative of the Taiwanese national profile specified in the 2016 Family and Fertility Survey Report [33], which might offset the drawbacks of the single-center study. Furthermore, our study attempted to collect information on the diet during pregnancy, but many study participants were unable to comply with this study aspect. Further research is needed to identify an appropriate method to collect relevant data to understand the influence of the nutrition-related impact as a confounding factor in analyses of air pollutant exposure and pregnancy outcomes. Lastly, we used data on ambient PM levels from TAQMN, EPA, which may not be exactly accurate for the personal exposure level. Current recommendations for the use of a personal environmental monitoring sampler to improve the detection of the exact exposure to PM_2.5_/PM_10_ might be a good research tool.

## 5. Conclusions

ETS may result in a 1.4 cm reduction in birth length. This relationship may reflect an independent risk factor for fetal growth, and pregnant women should avoid passive smoking in addition to exposure to PM. The urinary cotinine level might be applicable as a reliable objective parameter for measuring ETS exposure. Smoking in front of expectant mothers may heedlessly expose them to ETS. Public education should include strategies to counter indoor ETS. Childbirth education classes should include education on ETS within the home.

## Figures and Tables

**Table 1:** Characteristics of the maternal participants and neonates (*N* = 74).

Characteristics	*N* (%) or Mean ± Standard Deviation
Demographic characteristics of maternal participants	
Age, year	31.9 ± 4.2
Weight, kg	60.9 ± 10.8
Body mass index, kg/m^2^	23.6 ± 3.8
Education level, year	15.4 ± 1.7
Substance during pregnancy	
Alcoholic drinking (*n* = 67)	2 (3.0)
Nutritional supplements (*n* = 67)	60 (89.6)
Family smoking before pregnancy	36 (48.6)
subjective frequency of smoke exposure during pregnancy	
Number of smokers in home environment	2.0 ± 3.0
ETS exposure in past 30 days	3.0 ± 2.0
ETS exposure in past 7 days	2.0 ± 2.0
Perceived frequency of ETS exposure	2.0 ± 2.0
Objective exposure to smoke or air pollution	
Level of urinary cotinine at birth, ng/mL	4.7 ± 6.6 (range: 1.0–42.9)
Average PM_2.5_ level in the previous year, μg/m^3^	28.0 ± 2.3 (range: 18.2–31.7)
Average PM_10_ level in the previous year, μg/m^3^	48.4 ± 5.2 (range: 32.3–58.7)
Parameters of the newborns	
Gestational age, week	38.5 ± 0.8
Body weight, g	3033 ± 317
Head circumference, cm	33.4 ± 1.3
Length, cm	48.5 ± 1.8
Chest circumference, cm	32.1 ± 1.3

ETS = environmental tobacco exposure; PM = particulate matter.

**Table 2:** The relationship between categorical variables of exposure and newborn parameters (*N* = 74).

Variables	Gestational Week	Body Weight	Head Circumference	Length	Chest Circumference	Abnormal Appearance
Factor score of subjective exposure frequency to smoke (by tertile)						
Low (−1.3 to −0.5)	38.6 ± 0.8	3003.4 ± 344.0	33.4 ± 1.3	48.6 ± 2.0	32.3 ± 1.5	5 (23.8)
Moderate (−0.4 to 2.5)	38.7 ± 0.6	3104.4 ± 311.6	33.8 ± 1.5	48.8 ± 1.9	32.2 ± 1.2	4 (17.4)
High (2.6 to 3.4)	38.4 ± 1.0	3010.0 ± 302.6	33.0 ± 1.2	48.2 ± 1.7	31.8 ± 1.1	5 (25.0)
*p* value	0.419	0.502	0.170	0.526	0.416	0.806
Urinary cotinine, ng/mL						
None	38.6 ± 0.9	3046.1 ± 349.2	33.4 ± 1.3	48.5 ± 1.8	32.1 ± 1.4	7 (17.9)
Low (2.0–7.4)	38.4 ± 0.7	3079.3 ± 284.7	33.3 ± 1.2	49.1 ± 2.0	32.0 ± 1.2	3 (20.0)
High (7.5–42.9)	38.4 ± 0.9	2907.3 ± 251.6	33.1 ± 1.3	47.4 ± 1.4 ^ab^	31.9 ± 1.0	4 (33.3)
*p* value	0.685	0.338	0.816	0.049	0.869	0.518
PM_2.5_, μg/m^3^ (by median)						
<28.0	38.6 ± 0.8	3042.7 ± 339.4	33.3 ± 1.6	48.6 ± 1.8	32.0 ± 1.3	6 (25.0)
≥28.0	38.6 ± 0.6	3007.7 ± 267.1	33.4 ± 1.1	48.1 ± 1.8	32.0 ± 1.1	3 (12.0)
*p* value	0.960	0.692	0.920	0.289	0.893	0.240
PM_10_, μg/m^3^ (by median)						
<49.0	38.7 ± 0.8	2993.1 ± 320.3	33.1 ± 1.3	48.1 ± 1.6	31.8 ± 1.3	5 (19.2)
≥49.0	38.5 ± 0.7	3060.1 ± 293.1	33.6 ± 1.4	48.7 ± 2.0	32.2 ± 1.2	4 (17.4)
*p* value	0.526	0.444	0.165	0.239	0.179	0.868

PM = particulate matter; “^a^” indicates *p* < 0.05 vs. the “low group” and “^b^” indicates *p* < 0.05 vs. the “none or low” group.

**Table 3:** Newborn’s length on subgroup analysis by urinary cotinine level and subjective frequency of exposure to smoke/air pollution (*N* = 74).

	Urinary Cotinine, ng/mL			
Subgroup	None	Low (2.0–7.4)	High (7.5–42.9)	*p*-Value
Factor score of subjective exposure frequency to smoke (by tertile)				
Low (−1.3 to −0.5)	48.4 ± 2.0	50.7 ± 2.3	47.8 ± 1.0	0.136
Moderate (−0.4 to 2.5)	49.1 ± 2.0	49.3 ± 0.8	47.1 ± 2.1	0.098
High (2.6 to 3.4)	48.3 ± 1.4	48.1 ± 2.4	46.5 (n = 1)	0.623
PM_2.5_, μg/m^3^ (by median)				
<28.0	48.7 ± 1.8	49.3 ± 1.8	47.6 ± 2.0	0.495
≥28.0	48.1 ± 1.4	48.6 ± 2.2	47.4 ± 1.4	0.497
PM_10_, μg/m^3^ (by median)				
<49.0	48.3 ± 1.6	47.0 (*n* = 1)	46.7 ± 0.6	0.182
≥49.0	48.9 ± 1.9	48.9 ± 2.1	47.9 ± 1.8	0.520

PM = particulate matter.

**Table 4:** Multivariable linear regression analysis for the association between urinary cotinine level of maternal subject and newborn’s length.

Urinary Cotinine, ng/mL	Unadjusted		Adjusted *	
	*B* (95% CI)	*p*-Value	*B* (95% CI)	*p*-Value
None	Reference		Reference	
Low (2.0–7.4)	0.6 (−0.4 to 1.6)	0.257	0.6 (−0.4 to 1.6)	0.256
High (7.5–42.9)	−1.1 (–2.3 to −0.001)	0.049	−1.4 (−2.5 to −0.2)	0.021

*B* = unstandardized regression coefficient; CI = confidence interval; * Model adjusted for age, body mass index, subjective frequency of smoke exposure during pregnancy, nutritional supplements, and alcohol use of the maternal subjects.
